# IGF-1 and recovery in anorexia nervosa: evidence from population-based and clinical cohorts

**DOI:** 10.1210/clinem/dgag211

**Published:** 2026-05-24

**Authors:** Cristiano Dani, Livio Tarchi, Lorenzo Bonacchi, Sara Haydar, Emanuele Cassioli, Eleonora Rossi, Alessandra Fanelli, Francesco De Logu, Romina Nassini, Nadia Micali, Valdo Ricca, Giovanni Castellini

**Affiliations:** Department of Neuroscience, Psychology, Drug Research, and Child Health, University of Florence, 50139 Florence, Italy; Neuroscience and Sense Organs Department, Careggi University Hospital, 50134 Florence, Italy; Department of Neuroscience, Psychology, Drug Research, and Child Health, University of Florence, 50139 Florence, Italy; Neuroscience and Sense Organs Department, Careggi University Hospital, 50134 Florence, Italy; Department of Health Sciences, Center for Integrated Translational Research and Knowledge Transfer (INCLINE), University of Florence, 50139 Florence, Italy; Center for Eating and Feeding Disorders Research (CEDaR), Mental Health Center Ballerup, Copenhagen University Hospital-Herlev and Gentofte, 2750 Ballerup, Denmark; Department of Health Sciences, University of Florence, 50139 Florence, Italy; Neuroscience and Sense Organs Department, Careggi University Hospital, 50134 Florence, Italy; General Laboratory, Careggi University Hospital, 50134 Florence, Italy; Department of Health Sciences, Center for Integrated Translational Research and Knowledge Transfer (INCLINE), University of Florence, 50139 Florence, Italy; Department of Health Sciences, Center for Integrated Translational Research and Knowledge Transfer (INCLINE), University of Florence, 50139 Florence, Italy; Center for Eating and Feeding Disorders Research (CEDaR), Mental Health Center Ballerup, Copenhagen University Hospital-Herlev and Gentofte, 2750 Ballerup, Denmark; Institute of Biological Psychiatry, Mental Health Centre Sct Hans, Copenhagen University Hospital-Amager & Hvidovre, 4000 Roskilde, Denmark; Great Ormond Street Institute of Child Health, University College London, London WC1E 6BT, UK; Department of Health Sciences, University of Florence, 50139 Florence, Italy; Department of Health Sciences, University of Florence, 50139 Florence, Italy

**Keywords:** anorexia nervosa, insulin-like growth factor 1, IGF-1

## Abstract

**Context:**

Anorexia nervosa (AN) is associated with severe metabolic and endocrine alterations, including growth hormone (GH) resistance and reduced insulin-like growth factor 1 (IGF-1). The longitudinal behavior of IGF-1 during treatment remains incompletely characterized.

**Objective:**

To evaluate IGF-1 levels in current and weight-restored AN, determine metabolic correlates, and examine longitudinal changes during clinical treatment.

**Design:**

Cross-sectional analysis in a population-based cohort and longitudinal analysis in a clinical cohort.

**Setting:**

UK Biobank (UKB) and a specialist eating disorder clinic.

**Participants:**

(1) UKB: 129 adult women with current AN, 2380 weight-restored AN, and 2380 healthy controls (HC) matched for age, sex, and body mass index (BMI). (2) Clinical cohort: 189 adult women with AN assessed at baseline and 12-month follow-up.

**Main outcome measures:**

Plasma IGF-1 levels; secondary metabolic and reproductive hormones, including GH, insulin, glucose, free triiodothyronine (FT3), and gonadal hormones.

**Results:**

Across the UKB groups, IGF-1 levels showed a graded pattern: lowest in current AN, medium in weight-restored AN, and highest in HC. In the clinical cohort, IGF-1 correlated positively with insulin, glucose, and FT3, and negatively with GH, consistent with GH resistance. IGF-1 levels increased significantly over 12 months of treatment (*P* = .003), with higher BMI at baseline predicting greater increases. Higher IGF-1 levels were associated with a greater likelihood of menstrual function independent of BMI (*P* < .001).

**Conclusion:**

IGF-1 appears reduced in current AN and may only partly normalize with weight restoration. IGF-1 may reflect metabolic state and reproductive function, suggesting value as an indicator of severity and treatment response.

Anorexia nervosa (AN) is a serious psychiatric disorder characterized by a persistent and often chronic course (1), self-induced starvation, significant weight loss, and an intense fear of weight gain (2). Due to its early onset (3) and the high rate of medical and psychiatric comorbidities (4), AN represents a serious public health concern.

Individuals with AN experience a marked reduction in lean body mass, with a shift from the normal equilibrium of muscle protein synthesis and degradation toward a predominantly catabolic state (5). These individuals also show impaired bone formation, reduced bone mineral density, and a significantly elevated risk of fractures compared with healthy controls (HC) (6). Moreover, AN is associated with a range of endocrine disturbances, including hypothalamic amenorrhea (7), elevated cortisol levels (8), lower leptin levels (9), higher ghrelin levels (10), and resistance to growth hormone (GH), characterized by low circulating levels of insulin-like growth factor 1 (IGF-1) (5) than HC.

IGF-1 is an anabolic hormone, primarily produced by the liver in response to GH, that mediates GH effects and plays a crucial role in maintaining muscle mass and strength (11), while also exerting anti-apoptotic (12) and antioxidant functions (13). Elevated IGF-1 has been identified as a biomarker predicting Parkinson's disease risk (14), while alterations in the GH/IGF-1 axis have been linked to neurodegenerative and psychiatric disorders such as Alzheimer's disease, depression, and anxiety (15). Emerging evidence suggests that the GH/IGF-1 axis mediates the complex interaction between metabolic and inflammatory processes (16), and its secretion and function are regulated by pro-inflammatory cytokines such as tumor necrosis factor-α (TNF-α), interleukin-1β (IL-1β), and interleukin-6 (IL-6), highlighting its sensitivity to inflammatory states and contributing to insulin-resistance (17). These endocrine adaptations are part of a broader metabolic response to starvation, and recent consensus work from the World Federation of Societies of Biological Psychiatry (18) highlights the GH/IGF-1 axis and hypercortisolemia among the key candidate biomarkers reflecting illness-related metabolic dysregulation in AN. Within this framework, alterations of the GH/IGF-1 axis in AN may not only reflect nutritional deficiency and GH resistance, but also interact with key metabolic hormones such as leptin and ghrelin, and with pro-inflammatory pathways, thereby linking endocrine dysregulation to systemic inflammation (19), and increased cardiovascular vulnerability (20), as reduced IGF-1 levels may lead to loss of vascular protection and be associated with higher cardiovascular risk (21).

Recent findings have shown significantly lower IGF-1 levels in AN compared with HC (22), regardless of insulin resistance (23). However, no significant differences were observed between AN and those recovered from the disorder (24), suggesting a potential long-term alteration of the IGF-1 axis even after clinical recovery. Furthermore, IGF-1 reductions have been linked to impaired bone density in AN (25), pointing out how the impairment of its anabolic actions on osteoblast function and bone formation contributes to reduced bone mass and skeletal fragility in AN (26, 27). In the context of nutritional rehabilitation in AN, IGF-1 has been identified as a dynamic and responsive biomarker that reflects acute shifts from catabolic to anabolic nutritional status (28) and has also been shown to predict the resumption of menstrual function (29). Interestingly, while AN is associated with increased insulin sensitivity (30), circulating IGF-1 levels remain markedly reduced due to GH resistance and malnutrition (5), suggesting that, despite preserved insulin action, the loss of IGF-1 anabolic effects may contribute to catabolic processes and impaired recovery. IGF-1 regulation may also be influenced by genetic variants in metabolic pathways, as recent studies have identified variants associated with circulating IGF-1 and obesity traits (31).

Over the course of a 2-month treatment period, individuals with AN showed a significant increase in IGF-1 levels, which were positively associated with both body mass index (BMI) improvement and increasing leptin levels (32, 33). However, only a limited number of studies have explored the temporal dynamics of IGF-1 during active treatment using a long-term longitudinal design, particularly in adult populations (22).

Despite the well-documented role of IGF-1 in states of energy deprivation, its clinical translation in AN remains incomplete. As highlighted in a recent comprehensive meta-analysis (22), while cross-sectional studies consistently show reduced IGF-1 in AN, existing longitudinal data are largely limited to the early stages of refeeding and often suffer from small sample sizes. Therefore, the current literature lacks the robust evidence necessary to establish IGF-1 as a reliable biomarker of clinical outcome. This underscores the need for large-scale studies and extended longitudinal observations. To address these gaps, we first examined IGF-1 levels in individuals with AN compared with HC using data from the UK Biobank (UKB) (34). We then extended these findings in an independent, clinically recruited longitudinal cohort, with the aim of characterizing IGF-1 trajectories over a 12-month treatment period and investigating their correlation with anthropometric data, and metabolic and reproductive profiles (ie, menstrual function, as an index of reproductive axis reactivation during metabolic recovery).

## Materials and methods

### Study design

First, a cross-sectional analysis was conducted using data from the UK Biobank (UKB), a large prospective cohort providing data from approximately 500 000 individuals across the United Kingdom, aged between 40 and 69 years and recruited between 2006 and 2010 (35). We focused on UKB participants with an available serum IGF-1 measurement from the initial assessment visit (instance 0). Although a subset of participants also had IGF-1 measured at the first repeat assessment visit (instance 1), only instance 0 data were used in the present analyses to ensure consistency across the cohort. Within this group, we compared IGF-1 levels between individuals who met criteria for AN according to algorithmic phenotypes from the second Mental Health Questionnaire in UKB (MHQ2) (36), and HC. HC were selected from UKB female participants with available serum IGF-1 measurements who did not meet criteria for AN in MHQ2, did not report any other eating disorder (ED; eg, bulimia nervosa, binge-eating disorder), and had no ICD-coded ED in hospital records (F50.x). Additionally, participants with any other psychiatric conditions (self-reported, MHQ-based, or ICD-coded) were excluded. To ensure comparability, individuals with medical comorbidities known to affect IGF-1 (eg, thyroid disease, diabetes, chronic liver or kidney disease, chronic inflammatory conditions) were also removed.

Second, longitudinal analyses were conducted on individuals with AN recruited at the Outpatient Clinic for Eating Disorders of the Psychiatric Unit of the University of Florence, Italy. All diagnostic procedures were performed as part of a routine initial evaluation upon admission to the clinic. All participants were adequately informed about the study and provided written informed consent. The study protocol was approved by the Ethics Committee of the local institution (Comitato Etico Regione Toscana—Area Vasta Centro), and the study was conducted in accordance with the principles of the Declaration of Helsinki.

### Participants

We identified 2509 UKB participants of female sex with a self-reported history of AN based on the algorithmic phenotypes from the second Mental Health Questionnaire in UKB (MHQ2) (36) according to the Diagnostic and Statistical Manual of Mental Disorders, 5th Edition (DSM-5) criteria for AN (2), and with available serum IGF-1 measurements from the baseline visit (instance 0). Among these, we further divided the sample into 2 different groups: (1) individuals with a body mass index (BMI) ≤ 18.5 kg/m^2^, considered as individuals with current AN (C-AN), and (2) individuals with BMI > 18.5 kg/m^2^, considered as weight restored AN (WR-AN). The adopted threshold is consistent with the World Health Organization's definition of underweight and the International Classification of Diseases 11th Revision (ICD-11) (37) diagnostic guidelines for significantly low body weight in adult AN. This selection resulted in a final analytical sample of 129 individuals in C-AN group, and 2380 individuals in WR-AN group. The HC group consisted of 162 755 individuals of female sex with no documented history of ED or other psychiatric conditions, for whom IGF-1 data were also available. To ensure comparability with the WR-AN group, propensity score matching was applied. Propensity scores were estimated using logistic regression including age and BMI as covariates. Individuals were then matched 1:1 using nearest-neighbor matching without replacement, yielding a final HC sample of 2380 matched participants.

The clinical cohort was recruited at the outpatient unit of the Eating Disorder Center of the Department of Psychiatry at the University of Florence, provided they met the following inclusion criteria: (1) current diagnosis of AN, according to the DSM-5 (2); (2) female sex; (3) age >18 years; and (4) willingness to comply with the experimental procedures and to sign written informed consent. Exclusion criteria were as follows: comorbid schizophrenia, bipolar I disorder, acute psychotic disorder; current use of psychoactive medications (except for antidepressants and benzodiazepines); current or past use of medications known to interfere with bone or IGF-1 metabolism, such as bisphosphonates, glucocorticoids, hormone replacement therapies, or antiepileptic drugs; endocrine or metabolic disorders known to affect IGF-1 levels or bone metabolism, including untreated thyroid disease, diabetes mellitus, Cushing's syndrome, pituitary disease, and chronic renal failure; a history of bone fracture within the previous 6 months. Amenorrhea was defined as the absence of menstrual bleeding for ≥3 consecutive months in individuals with previously regular menstrual cycles, or ≥6 months in those with previously irregular cycles, in accordance with standard clinical criteria for functional hypothalamic amenorrhea (38). At baseline, 160/189 (84.7%) individuals presented with amenorrhea. After 12 months of follow-up, 19 of these individuals had regained a stable menstrual function for at least 3 consecutive months, corresponding to a 12% recovery rate among those who were amenorrheic at baseline.

### Biomarker determinations

Sampling procedures and biomarker quantification protocols for the UKB samples are described in detail elsewhere (35).

For the clinical cohort, blood samples were collected in the morning between 08:00 and 10:00 after an overnight fast. For IGF-1 quantification, blood was drawn into EDTA tubes; plasma was obtained by centrifugation at 3000 rpm for 10 minutes at 4 °C, aliquoted, and stored at −80 °C until analysis. Plasma IGF-1 concentrations were measured using an electrochemiluminescence immunoassay on the Cobas e-series platform (Elecsys IGF-1, Roche Diagnostics, Mannheim, Germany), following the manufacturer's instructions. For all other hormonal and metabolic parameters, blood was collected in serum separator tubes. Serum was separated by centrifugation, aliquoted, and stored at −80 °C prior to quantification. Serum concentrations of GH, thyroid-stimulating hormone (TSH), free triiodothyronine (FT3), free thyroxine (FT4), and insulin were measured using Roche Elecsys electrochemiluminescence immunoassays on the same Cobas e-series platform. Reproductive hormones, including luteinizing hormone (LH), follicle-stimulating hormone (FSH) (17), estradiol (E2), progesterone, testosterone, and sex hormone–binding globulin (SHBG), were also quantified using automated immunoassays (Cobas e-series, Roche Diagnostics). All assays were performed in accordance with the manufacturer's guidelines.

### Data analyses

Continuous variables were reported as mean and standard deviation. Comparisons between individuals with AN and HC in the UKB cohort were performed using analysis of covariance (ANCOVA) to examine differences in serum IGF-1 levels, adjusting for age and BMI. Resulting *P*-values were corrected using Tukey's criterion for multiple comparisons. Baseline differences in LH levels between subgroups (amenorrhea vs eumenorrhea) were analyzed using the Wilcoxon rank-sum test. Moreover, partial Spearman's rank correlation coefficients, adjusted for age and BMI, were calculated to examine the associations between metabolic parameters. To correct for the increased risk of false positives due to multiple comparisons, a Bonferroni correction was applied to the *P*-values. Longitudinal changes in IGF-1 levels were analyzed using linear mixed-effects models with random intercepts. These models account for individual-level variability and allow the evaluation of within-subject changes over time while controlling for baseline heterogeneity. Age and BMI were included as covariates to adjust for potential confounding effects. To address the hierarchical structure of the data, nested models were specified, incorporating patient identifiers as grouping factors.

An a priori power analysis was conducted based on repeated-measures ANOVA assumptions (2 time points, correlation among repeated measures = 0.40, nonsphericity correction *ε* = 1). Assuming a small-to-moderate effect size (*f* = 0.20), an alpha level of 0.05, and a desired power of 0.95, the analysis indicated that a total sample size of 100 participants would be required to reliably detect longitudinal changes in IGF-1 levels.

To investigate determinants of menstrual status (amenorrhea vs eumenorrhea), we estimated generalized linear mixed-effects models with a binomial logit link. Menstrual status was operationalized as a binary outcome (0 = amenorrhea, 1 = eumenorrhea). IGF-1 levels were specified as the primary predictor, while age and BMI were included as covariates to adjust for potential confounding. A random intercept at the participant level accounted for within-subject dependency arising from repeated measurements over time. To evaluate whether the relationship between IGF-1 and menstrual status changed longitudinally, an interaction term between IGF-1 and time (days since baseline) was included. Model coefficients were exponentiated to obtain adjusted odds ratios (ORs) and corresponding 95% confidence intervals (CIs).

Sensitivity analyses were conducted using ANCOVA models to account for potential confounders not included in the main analysis. These models adjusted for inflammatory status (C-reactive protein levels [CRP]; mg/dL), 24-hour caloric intake (kj), antidepressant use, body composition parameters (body fat mass, whole body fat mass, and whole body water mass, smoking status). Due to the structure of the UKB dataset, these analyses were performed on the subsets of participants with available data for each specific variable.

All statistical analyses were performed using R 4.5.1 (*RStudio Team. RStudio: Integrated Development for R.*, 2020) and the following packages: nlme (39), dplyr (40), ggplot2 (41).

## Results

### UK Biobank

The comparison of IGF-1 levels across groups in the UKB sample revealed a significant main effect (C-AN vs WR-AN vs HC) of group after adjusting for age and BMI (*P* < .001). In the overall model, age showed a strong association with IGF-1 (F = 518.36, *P* < .001), whereas BMI did not account for additional variance (*F* = 3.24, *P* = .07). Post-hoc Tukey tests indicated that C-AN had significantly lower IGF-1 levels compared to both WR-AN (*P* = .002) and HC (*P* < .001). Furthermore, WR-AN exhibited significantly reduced IGF-1 levels relative to HC (*P* = .015). Results are shown in Table 1.

**Table 1:** Group comparisons across the UKB sample

	C-AN (*n* = 129)	WR-AN (*n* = 2380)	HC (*n* = 2380)	F	Post-hoc Tukey
Age	51.73 ± 7.24	51.26 ± 6.81	51.01 ± 7.80	1.14	n.s.
BMI (kg/m^2^)	17.48 ± 0.98^a^	22.97 ± 3.90^b^	23.04 ± 3.47^b^	144.30***	a < b
IGF-1 (nmol/L)	19.80 ± 5.07^a^	21.70 ± 5.69^b^	22.21 ± 5.77^c^	11.10***	a < b < c

*F*-values alongside their *P*-values and Tukey's post-hoc analysis are shown. WR-AN and HC were matched 1:1 using nearest-neighbor matching without replacement, based on age and BMI. Superscript letters indicate Tukey’s post-hoc groupings within each row. Values with different letters are significantly different from each other; values with the same letter are not significantly different. *** *P* < .001.

Abbreviations: BMI, body mass index; C-AN, current anorexia nervosa; HC, healthy controls; IGF-1, insulin-like growth factor 1; WR-AN, weight restored anorexia nervosa.

### Clinical sample

Baseline characteristics of the clinical sample are summarized in Table 2. Baseline LH levels were significantly lower in individuals with amenorrhea compared to those in eumenorrhea (median 1.25 vs 5.70 mIU/mL; *W* = 997, *P* < .001). Several significant partial correlations were observed between IGF-1 and hormonal and metabolic parameters, after adjusting for age and BMI (Fig. 1). After Bonferroni correction for multiple comparisons, IGF-1 levels were positively associated with insulin (*ρ* = 0.40, *P* < .001), glucose (*ρ* = 0.29, *P* = .001), and FT_3_ (*ρ* = 0.35, *P* = .0001), suggesting an interaction between the somatotropic axis and both metabolic and thyroid function. Overall, metabolic and hormonal biomarkers showed expected physiological coupling, including positive correlations among thyroid hormones and glucose-insulin measures, and inverse correlations between GH and both IGF-1 and insulin. No other significant correlations were detected among the remaining parameters.

**Figure 1:**
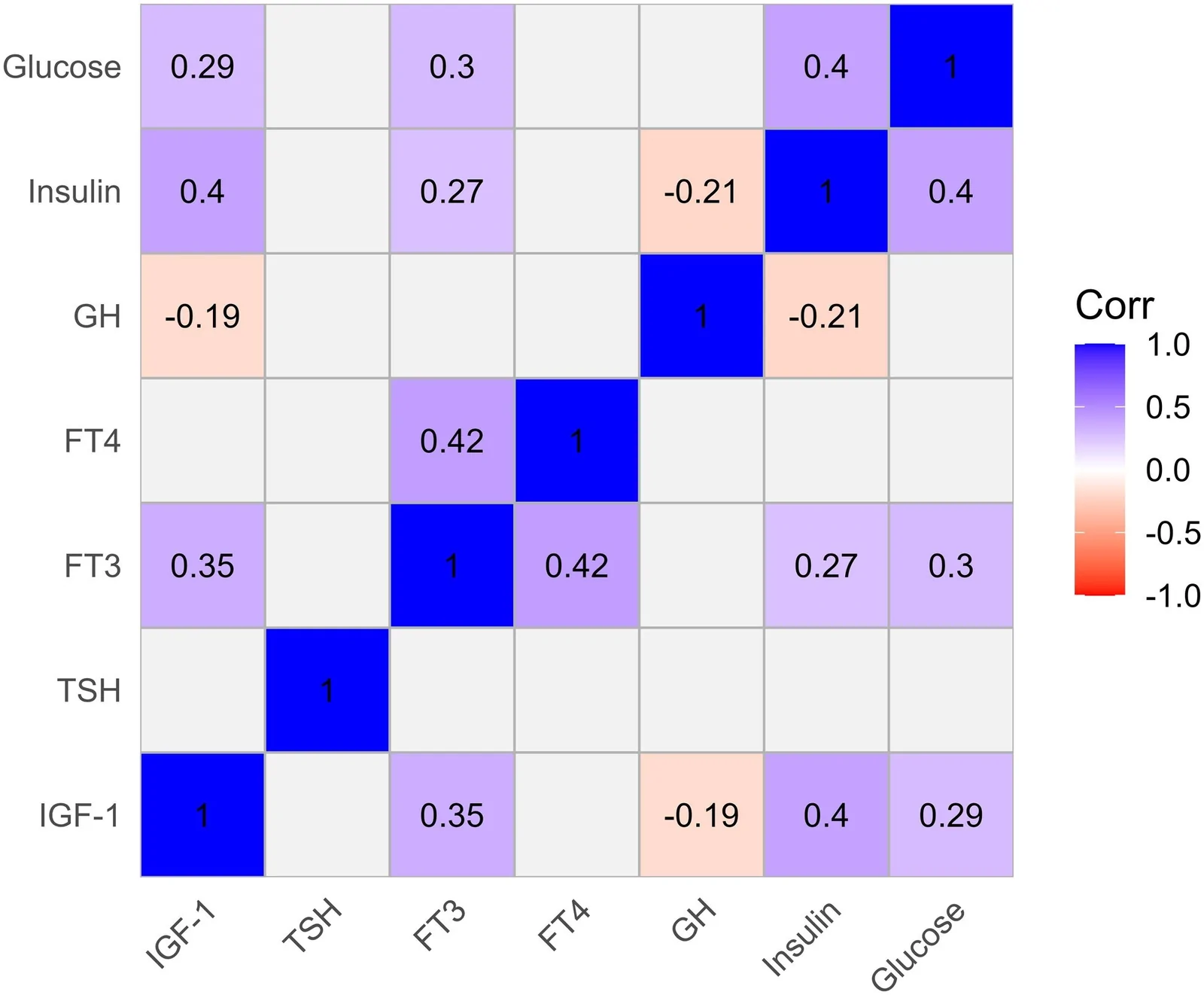
Partial spearman's rank correlation matrix of metabolic and endocrine parameters. The heatmap displays partial Spearman's correlation coefficients (*ρ*) among hormonal and metabolic parameters at baseline, adjusted for age and BMI. Only correlations significant after Bonferroni correction for multiple comparisons (*P* < .05, Bonferroni-adjusted) are shown. Positive correlations are indicated in shades of blue, and negative correlations in shades of red, with color intensity representing the strength of the correlation (range: −1 to +1). White cells indicate correlations that are not significant after Bonferroni correction. *Abbreviations:* FT3, free triiodothyronine; FT4, Free thyroxine; GH, growth hormone; IGF-1, insulin-like growth factor 1; TSH, thyroid-stimulating hormone.

**Table 2:** Baseline descriptives of the clinical sample

	AN (n = 189)	Follicular phase range
Age	23.73 ± 8.59	—
BMI (kg/m^2^)	15.5 ± 1.41	—
Duration of illness (age)	6.35 ± 8.75	—
IGF-1 (nmol/L)	21.32 ± 9.03	—
GH (μg/L)	4.32 ± 5.94	—
Insulin (mIU/L)	5.82 ± 5.63	—
Glucose (mg/dL)	70.63 ± 13.98	—
TSH (μIU/mL)	1.89 ± 1.09	—
Free T_3_ (pmol/L)	3.55 ± 1.08	—
Free T_4_ (pmol/L)	13.33 ± 2.29	—
E2 (pg/mL)	30.35 ± 84.57	12.4-233
FSH (mIU/mL)	8.45 ± 18.31	3.5-12.5
LH (mIU/mL)	5.39 ± 8.87	2.4-12.6
Progesterone (ng/mL)	2.81 ± 14.65	0.06-0.89
Testosterone (nmol/L)	0.85 ± 0.52	—
SHBG (nmol/L)	116.66 ± 80.49	—

Baseline median LH was 1.25 mIU/mL in individuals with amenorrhea (*n* = 160) and 5.70 mIU/mL in those with eumenorrhea (*n* = 29).

Abbreviations: E2, (17)estradiol; FSH, follicle-stimulating hormone; GH, growth hormone; IGF-1, insulin-like growth factor 1; LH, luteinizing hormone; SHBG, sex hormone–binding globulin; T_3_, triiodothyronine; T_4_, thyroxine; TSH, thyroid-stimulating hormone.

### Longitudinal patterns

The longitudinal analyses of IGF-1 levels revealed a significant increase at 12 months follow-up in individuals with AN (Std. *β* = .17, *P* = .003), after adjustment for age and BMI at baseline. Mean IGF-1 levels increased from T0: 21.32 ± 9.03 nmol/L to T12: 26.55 ± 5.85 nmol/L. The linear mixed-effects model demonstrated that time in treatment was a significant predictor of IGF-1 increase independent of BMI. Concurrently, higher BMI was independently associated with higher IGF-1 levels (Std. *β* = .19, *P* = .005), while older age predicted lower IGF-1 levels (Std. *β* = −.34, *P* < .001). Illness duration was not significantly associated with IGF-1 levels (Std. *β* = −.17, *P* = .289). Figure 2 illustrates the estimated trajectory of IGF-1 increase over the 12-month follow-up.

**Figure 2:**
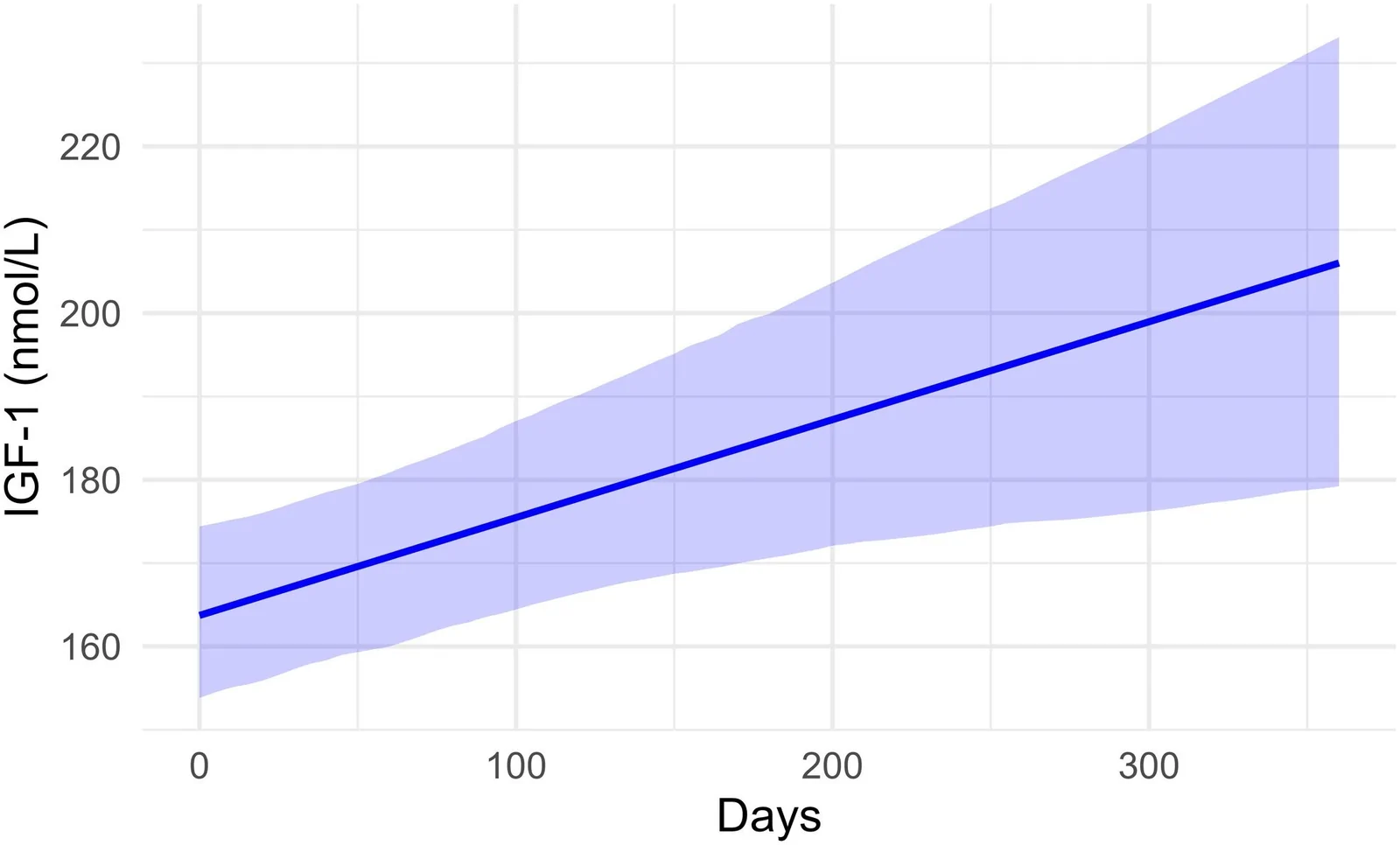
Longitudinal changes in IGF-1 levels (ng/mL) over time (days). The blue line represents the estimated trajectory from the linear mixed-effects model, and the shaded area indicates the 95% confidence interval. *Abbreviations:* IGF-1, insulin-like growth factor 1.

### IGF-1 and reproductive function

The generalized linear mixed-effects model in the clinical sample, adjusted for age and BMI, showed that higher IGF-1 levels were significantly associated with a greater likelihood of eumenorrhea (adjusted OR = 1.04, 95% CI 1.04-1.05, *P* < .001). Higher BMI was also associated with eumenorrhea (adjusted OR = 1.50, 95% CI 1.50-1.51, *P* < .001). Older age was associated with a lower likelihood of eumenorrhea, indicating poorer menstrual function with increasing age (adjusted OR = 0.997, 95% CI 0.994-1.000, *P* = .031).

After Bonferroni correction for multiple comparisons, several significant partial correlations among IGF-1 and reproductive hormones were observed at baseline after adjusting for age and BMI (Fig. 3). IGF-1 was positively associated with LH (*ρ* = 0.30, *P* < .001) and E2 (*ρ* = 0.33, *P* = .014). In contrast, IGF-1 showed a negative correlation with SHBG (*ρ* = −0.29, *P* = .028).

**Figure 3:**
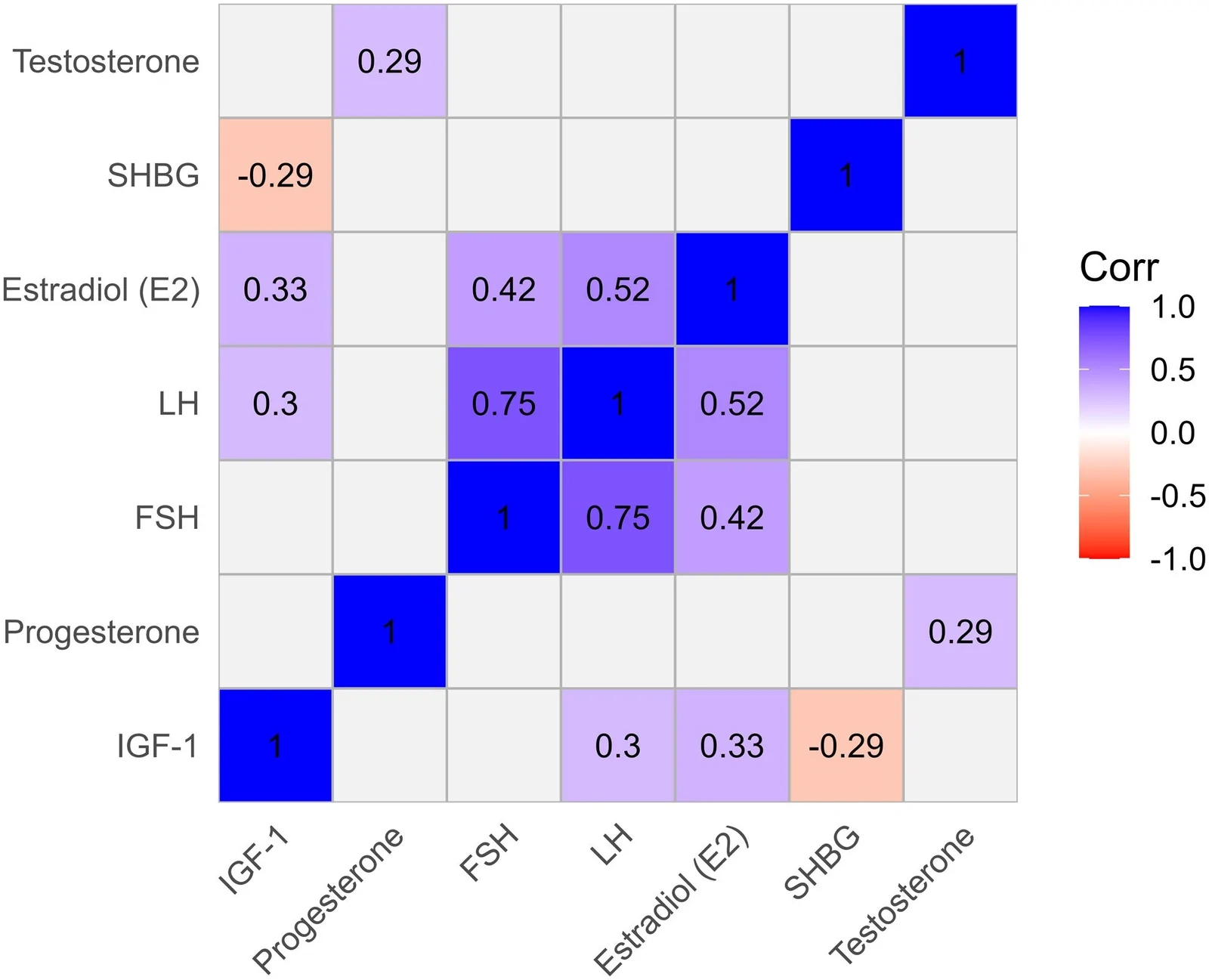
Partial spearman's rank correlation matrix of IGF-1 and reproductive hormones. The heatmap displays partial Spearman's correlation coefficients (*ρ*) among IGF-1 and reproductive hormones at baseline, adjusted for age and BMI. Only correlations significant after Bonferroni correction for multiple comparisons (*P* < .05, Bonferroni-adjusted) are shown. Positive correlations are indicated in shades of blue, and negative correlations in shades of red, with color intensity representing the strength of the correlation (range: −1 to +1). White cells indicate correlations that are not significant after Bonferroni correction. *Abbreviations:* E2, (17)estradiol; FSH, follicle-stimulating hormone; IGF-1, insulin-like growth factor 1; LH, luteinizing hormone; SHBG, sex hormone–binding globulin.

The gonadotropins displayed the expected coordinated pattern: LH and FSH were correlated with one another (*ρ* = 0.75, *P* < .001), and both were positively correlated with E2 (LH: ρ = 0.52, *P* < .001; FSH: *ρ* = 0.42, *P* < .001). Progesterone was positively correlated with testosterone (*ρ* = 0.29, *P* = .022). No additional correlations met the threshold for significance following Bonferroni correction.

### Sensitivity analyses

Sensitivity analyses (ANCOVA) confirmed that the main effect of group on IGF-1 levels remained significant after adjusting for CRP levels (*P* < .001), antidepressant use (*P* < .001), recurrent episodes of overeating/binge-eating (*P* = .002), body fat mass (*P* < .001), whole body fat mass (*P* < .001), whole body water mass (*P* < .001), and smoking status (*P* < .001). Group effect did not reach statistical significance when adjusting for 24-hour caloric intake (*P* = .180). Results are reported in Table S1 (42).

## Discussion

The present study provides new insights into the dynamics of IGF-1 in AN, integrating evidence from a large population-based dataset (UKB) with a clinically recruited longitudinal sample. This dual approach allowed us to confirm cross-sectional alterations of the somatotropic axis in AN and to characterize the temporal trajectory of IGF-1 during treatment. Consistent with previous literature, we observed significant alterations in serum IGF-1 levels in AN compared with HC, as well as a longitudinal increase during the course of treatment (22).

Specifically, individuals with C-AN in the UKB sample displayed significantly lower IGF-1 serum levels when compared with both WR-AN and HC, and significantly lower in WR-AN than HC. These results are in line with previous findings, highlighting that individuals with AN exhibit lower IGF-1 levels than HC during the acute phase (22, 24). Adopting a weight restoration-based perspective, only a partial normalization of IGF-1 levels was observed, which may indicate a sub-threshold GH resistance resulting from prolonged starvation. By confirming this well-established pattern in a large, independent, population-based dataset, our findings extend the generalizability of prior clinical studies, which were often limited to relatively small cohorts (22).

Findings from the clinical cohort provided, for the first time in AN, a comprehensive overview of the metabolic feedbacks involving the somatotropic axis (5). The negative correlation of IGF-1 with GH may reflect the well-known state of GH resistance of individuals with AN, whereby elevated GH secretion fails to translate into adequate hepatic IGF-1 production (5). This alteration may be partly explained by down-regulation of GH receptor expression, in agreement with reduced GH binding protein levels (23, 43). Notably, IGF-1-independent effects of GH on lipolysis and gluconeogenesis remain preserved, as also shown in Pappa2-deficient mice, where elevated GH levels were associated with enhanced lipid mobilization and impaired hepatic lipogenesis (44). This adaptation allows GH to maintain euglycemia and mobilize energy substrates under severe energy deprivation (45). The altered feedback between IGF-1 and GH in AN may also be influenced by other endocrine adaptations, such as higher fibroblast growth factor-21 (46), elevated ghrelin (10), reduced leptin (9), and hypercortisolemia (8).

Furthermore, the positive correlations of IGF-1 with insulin and glucose may indicate that IGF-1 remains linked to residual metabolic status, even if in the context of impaired metabolic function due to energy deprivation (47), in line with recent evidence showing that individuals with AN spend a substantial proportion of the day in prolonged hypoglycemia (48). The positive correlation of IGF-1 with FT3 also suggests an integrated downregulation of both the somatotropic and thyroid axes in AN. Previous evidence showed that FT3 is significantly reduced in AN, but not in constitutional thinness, where levels remain similar to HC (49). This suggests that low FT3 may be a specific endocrine response to energy deprivation in AN, alongside IGF-1 suppression. In our cohort, IGF-1 was positively associated with FT3, but not with TSH or FT4. This pattern reflects the low-T3 syndrome typical of AN, likely driven by reduced T4-to-T3 conversion and a hypothalamic-pituitary adjustment that maintains TSH in the low-normal range (50).

The longitudinal analyses demonstrated a significant increase in IGF-1 serum levels in individuals with AN over 12 months of treatment follow-up. These findings align with previous longitudinal studies (32, 33) and extend this evidence by confirming the associations in a larger cohort. The progressive rise in IGF-1 and its positive association with higher BMI levels may confirm the shift from a catabolic to an anabolic state, highlighting the role of IGF-1 as a sensitive biomarker of metabolic recovery (28). Notably, age confirmed as a negative predictor of IGF-1 levels (51), indicating that age-related factors may influence the restoration of the somatotropic axis in AN, independently of illness duration. However, the persistence of this association after age-adjustment further supports the notion that impaired IGF-1 levels may represent a disorder-specific metabolic trait in AN.

The observed reduction in IGF-1 levels in AN likely reflects a state of GH resistance secondary to chronic energy deficiency, contributing to impaired gonadotropin-releasing hormone (GnRH) pulsatility and subsequent reproductive dysfunction, as described in models of functional hypothalamic amenorrhea (52). In line with this model, in our clinical sample, higher IGF-1 levels were independently associated with regular menstrual function, even after adjustment for age and BMI, supporting the notion that IGF-1 serves as a metabolic permissive signal for the activation of the reproductive axis (53). Specifically, IGF-1 may facilitate this activation by the stimulation of GnRH neuronal activity and gene expression (53), while simultaneously upregulating Kiss1 expression to provide the necessary kisspeptin-mediated excitatory input (54), and potentially enhancing the sensitivity of pituitary gonadotrophs to GnRH pulses (53).

Taken together, the observed associations indicate that regular menstrual function in AN may depend not solely on body weight, but also on the degree of anabolic recovery, as reflected by IGF-1 levels (53, 55). This interpretation may be further supported by hormonal correlations observed at baseline: higher IGF-1 levels aligned with biomarkers of hypothalamic-pituitary-ovarian activity may be consistent with IGF-1 acting as a metabolic signal enabling the restoration of GnRH and gonadotropin release (56). The inverse correlation between IGF-1 and SHBG likely reflects the nutritional regulation of hepatic SHBG synthesis, which increases under conditions of energy deficiency—such as AN—and declines as anabolic tone and IGF-1 signaling improve, consistent with experimental evidence linking SHBG expression to hepatic metabolic status and IGF-1-mediated regulation (57, 58). The coordinated pattern among LH, FSH, and E2 aligned with expected feedback dynamics, while the links between progesterone and androgen precursors indicate preserved steroidogenic pathways despite hypothalamic suppression (38). Overall, these data emphasize that endocrine normalization in AN involves more than simple weight restoration, requiring the re-establishment of metabolic equilibrium.

More broadly, our findings highlight IGF-1 as a clinically informative biomarker in AN, reflecting both the degree of metabolic suppression and the trajectory of recovery. Its associations with BMI and menstrual function support its role in monitoring anabolic shift during treatment, while persistently low levels may signal risk for incomplete remission. Given its involvement in bone fragility (25), cardiovascular vulnerability (21), and impaired endocrine functions (5), IGF-1 could aid in stratifying disease severity and tailoring clinical follow-up (59).

However, several limitations should be acknowledged. First, in the UKB cohort, our AN definition relied on the algorithmic phenotype derived from self-reported questionnaire data (MHQ2) (36). IGF-1 was available from both the initial assessment visit (instance 0) and, for a subset of participants, the first repeat assessment visit (instance 1); however, only baseline measurements were used in this study, precluding examination of temporal fluctuations. In addition, specific clinical details such as duration of amenorrhea, duration of illness, AN subtype, history of treatment, and use of benzodiazepines were not consistently available in the UKB cohort, preventing a comprehensive evaluation of their potential influence on IGF-1 levels. Furthermore, while sensitivity analyses were conducted to account for key confounders, they were restricted to subsets of participants with available data. Second, UKB participants are predominantly of Caucasian ancestry, which may limit the generalizability of the findings to other ethnic groups. Third, the clinical cohort was restricted to adult Caucasian females, which limits the generalizability of our findings to males, adolescents, and individuals from other ethnic backgrounds. Moreover, the follow-up was limited to 12 months, preventing evaluation of long-term IGF-1 trajectories beyond weight restoration. While this timeframe provides valuable longitudinal data on the acute phase of rehabilitation, it may not be sufficient to capture the complete long-term normalization of the somatotropic axis or the full recovery of reproductive function for all patients. Finally, although correlations with insulin, glucose, and thyroid hormones highlight physiological coupling, causality cannot be inferred from observational data. Future research should extend follow-up durations to determine whether IGF-1 normalization persists and whether residual deficits predict relapse or long-term complications. Integration of genetic and inflammatory biomarkers could clarify the mechanisms linking GH resistance, somatotropic axis suppression, and metabolic adaptation.

In conclusion, this study confirmed that IGF-1 levels are markedly reduced in individuals with AN compared with HC and demonstrated a progressive rise during treatment, closely associated with BMI and metabolic recovery. By combining cross-sectional and longitudinal data, our findings reinforce the role of IGF-1 as a dynamic biomarker of the catabolic-to-anabolic transition in AN. Monitoring IGF-1 may therefore provide clinically relevant information to evaluate treatment response and guide personalized therapeutic strategies (60).

## Data Availability

The data that support the findings of this study are available from UKBioBank. Restrictions apply to the availability of these data, which were used under licence for this study. Data are available from https://www.ukbiobank.ac.uk/ with the permission of UKBioBank. This research has been conducted using the UK Biobank Resource under Application Number 501518. The data from the clinical cohort supporting the findings of this study are available from the corresponding author upon reasonable request and with the agreement of all co-authors.
